# Visual loss in HIV-associated cryptococcal meningitis: A case series and review of the mechanisms involved

**DOI:** 10.4102/sajhivmed.v16i1.305

**Published:** 2015-10-16

**Authors:** Anand Moodley, William Rae, Ahmed Bhigjee

**Affiliations:** 1 Department of Neurology, Greys Hospital, South Africa; 2 Department of Neurology, University of KwaZulu-Natal, South Africa; 3 Department of Medical Physics, University of The Free State, South Africa

## Abstract

Permanent visual loss is a devastating yet preventable complication of cryptococcal meningitis. Early and aggressive management of cerebrospinal fluid pressure in conjunction with antifungal therapy is required. Historically, the mechanisms of visual loss in cryptococcal meningitis have included optic neuritis and papilloedema. Hence, the basis of visual loss therapy has been steroid therapy and intracranial pressure lowering without clear guidelines. With the use of high-resolution magnetic resonance imaging of the optic nerve, an additional mechanism has emerged, namely an optic nerve sheath compartment syndrome (ONSCS) caused by severely elevated intracranial pressure and fungal loading in the peri-optic space. An improved understanding of these mechanisms and recognition of the important role played by raised intracranial pressure allows for more targeted treatment measures and better outcomes. In the present case series of 90 HIV co-infected patients with cryptococcal meningitis, we present the clinical and electrophysiological manifestations of Cryptococcus-induced visual loss and review the mechanisms involved.

## Introduction

Meningitis owing to *Cryptococcus neoformans* remains a frequent human immunodeficiency virus (HIV)- associated opportunistic infection even in developing countries with effective antiretroviral therapy (ART) rollout programs.^1^ This is largely a result of failure of HIV testing by individuals with risky sexual behaviour, and late presentation for and poor compliance with ART. Therefore, it is not uncommon to still encounter severely immunocompromised patients presenting for the first time with opportunistic infections and CD4+ T-lymphocyte counts < 100 cells/µL. Headache, high fever, nuchal pain and stiffness, photophobia, confusion, nausea, vomiting and diplopia are the common presenting symptoms of cryptococcal meningitis (CM). Symptoms arise from raised intracranial pressure and meningeal inflammation, usually within 1–2 weeks of the onset of the illness. High cerebrospinal fluid (CSF) pressure, depressed level of consciousness and an acellular CSF are poor prognostic features. Effective antifungal therapy (amphotericin B, flucytosine and fluconazole) is not readily available in most developing countries.^2^ Mortality remains high and contributes up to 20% of HIV-related deaths.^1^ Complications in survivors are severe, with visual loss being the most disabling, yet are potentially preventable and reversible. Recognition of visual impairment in encephalopathic patients is difficult and therefore often neglected and underreported. In the following case series, we evaluated 90 patients with culture-confirmed CM. Their results and a discussion of the mechanisms implicated in Cryptococcus-induced visual loss are discussed. An illustrative case of the optic nerve sheath compartment syndrome (ONSCS) as a putative mechanism is also presented in the discussion.

## Method

In a prospective study approved by the Greys Hospital and University of KwaZulu-Natal Ethics Committees, we consecutively recruited 90 patients with culture-confirmed CM between February 2008 and December 2011 (Table 1). Patients with reduced levels of consciousness were excluded (GCS < 14). All were HIV co-infected, provided informed consent, had full neuro-ophthalmological assessments and had magnetic resonance imaging (MRI) using standard imaging protocols. Patients were recruited within 4 weeks of the disease onset, during the induction and consolidation phases of CM treatment. Drug treatment and management of raised intracranial pressure were based on the 2007 South African HIV Clinician Society Guidelines.^3^

**TABLE 1:** Demographic data, cerebrospinal fluid pressure and CD4 count of 90 cryptococcal meningitis patients.

Patient characteristics	Patient data
Age in years: mean (range)	33.5 (18–51)
Male: *n* (%)	50 (55.6%)
CD4 count in cells/µL: mean (s.d.)	47 (10.1)
On ART: *n* (%)	22/90 (24.4%)
CSF pressure in cm CSF: mean (s.d.)	31.3 (13.5)

s.d., standard deviation; ART, antiretroviral therapy; CSF, cerebrospinal fluid.

Eighty-six patients underwent electrophysiological testing that involved visual evoked potentials (VEP) and Humphreys visual fields (HVF). VEP involved testing of each optic nerve's functioning by requesting the patient to look at a screen one metre away that displayed an alternating full-field checkerboard pattern. The cortical responses thus obtained were detected by silver-surface electrodes placed over the occipital scalp. Averaging of the cortical responses provided a reliable and reproducible triphasic wave from which the P100 latency (the large positive wave that occurs at approximately 100 ms from the stimulus) and amplitude (the vertical height in µV between the largest positive P100 and negative N80 waves) were obtained in accordance with International Society for Clinical Electrophysiology of Vision (ISCEV) guidelines.^4^ HVF was performed using the SITA 30-2 standard protocol. Pattern deviation fields that fulfilled acceptable reliability indices were included for analysis. Flash VEP using LED goggles were used in patients who were delirious; however, HVF was not possible in such patients.

### Statistics

Visual acuity, VEP latency and amplitude were dichotomised into abnormal and normal groups using standard normal references. One-sample *t* tests were used to compare mean latency and amplitude with laboratory references that have been previously described.^5^ Tests for association between groups were analysed using a chi-square test or Fisher's exact test, as appropriate. Statistical analysis was done by STATA, version 12.

## Results and discussion

### Clinical findings

Visual loss occurred at any stage of the illness and occurred frequently before starting drug therapy. Subgroup analysis not reflected in Table 2 showed that the majority of cases occurred within 2–4 weeks of CM onset, regardless of drug therapy. Rex's landmark article in 1993 of Cryptococcus-induced visual loss suggested two main mechanisms: an early and sudden visual loss owing to optic neuritis, and a late and gradual visual loss owing to papilloedema. Such distinct mechanisms, however, do not exist in isolation and an explanation for visual loss where neither mechanism is in operation needs clarification.^6^ Gradual, symmetrical and bilateral visual blurring associated with headache was the most common presentation in our series (Table 2). Sudden and catastrophic visual loss was rare, occurring in only one patient. Forty-six percent of patients had appreciable loss of vision (< 6/9 on Snellen) and profound visual loss of < 6/60 in 13%. Colour desaturation, pupillary reflex changes and pain on eye movement were relatively uncommon. Sixth nerve palsies owing to elevated intracranial pressure or meningitis occurred in 16% of patients. Bilateral and symmetrical cerebellar ataxia was common in this group and probably accounted for the impaired smooth pursuit and nystagmus – findings also commonly encountered in HIV-associated neurocognitive disorder.

**TABLE 2:** Neuro-ophthalmological manifestations of cryptococcal meningitis in 90 patients.

Examination parameter	Clinical findings	Proportion examined	
		Proportion	%
Best corrected visual acuity (Snellen)	< 6/9	41/90	46
	< 6/60	12/90	13
Mode of onset of visual loss	Bilateral/unilateral	34/41; 7/41	83; 17
	< 1 week	6/41	15
	> 1 week	35/41	85
	Sudden	1/41	2
	Pain on eye movement	1/41	2
	Colour desaturation	5/41	12
External ophthalmoplegia	Bilateral 6th nerve palsy	7/90	8
	Unilateral 6th nerve palsy	7/90	8
	Unilateral 3rd nerve palsy	1/90	1
Supranuclear eye movements	Impaired smooth pursuit	23/90	26
	Gaze-evoked nystagmus	20/90	22
	Convergence spasm	1/90	1
Swollen optic disc	Bilateral	26/90	29
	Unilateral	3/90	3
Pale optic disc	Bilateral and mild	2/90	2
Pupillary reflex	Reactive but sluggish	11/90	12
	No reaction	4/90	4
	RAPD	5/90	6

RAPD, relative afferent pupillary defect.

### Electrophysiological findings

**FIGURE 1:**
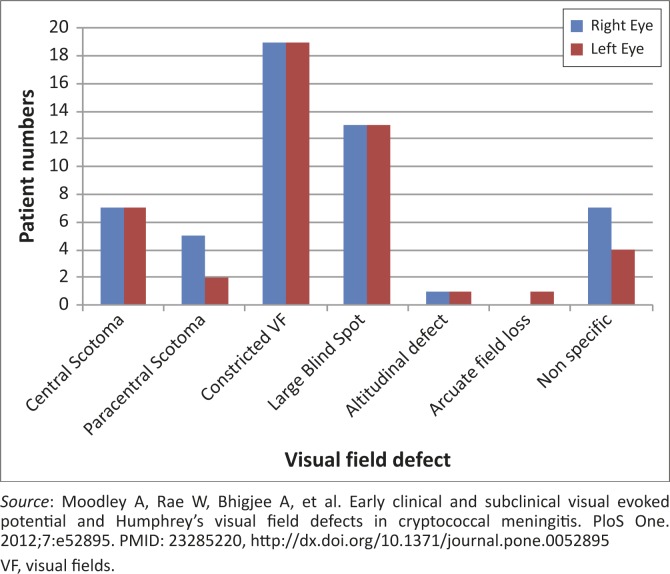
Frequencies of visual field defects.

VEP testing and HVF defects were common in the series we reported, both in visually impaired and visually normal patients with CM (Table 3).^5^ In the cross-section of 86/90 patients who underwent electrophysiological tests, VEP abnormalities were detected in visually impaired patients (68.9% of right eyes and 67.6% of left eyes), and in visually normal patients (56.5% of all eyes). In subgroup analysis, prolongation of the P100 latency was the predominant abnormality (42.3% of all eyes).^5^ In the absence of demyelination, these findings were interpreted as resulting from conduction block caused by optic nerve compression. Optic nerve compression with secondary conduction block and optic nerve infiltration were both deemed likely from these findings. VEP amplitude changes suggesting axonal loss were less frequent (14.6%) in eyes tested. As shown in Table 3, HVF abnormalities were also very frequent in patients who could be tested (76.6% of right eyes and 71.1% of left eyes). The predominant field defects were peripheral constriction with large blind spots – field defects consistent with papilloedema-related optic nerve dysfunction (Figure 1).^5^ Consequently, the interpretation of these findings was that the HVF defects supported raised intracranial pressure as an important cause of optic nerve dysfunction in Cryptococcus-induced visual loss.

**TABLE 3:** Frequencies of abnormal visual acuity, visual evoked potentials and Humphreys visual fields in 86 patients tested.

Findings	Visual acuity: < 6/9		VEP		HVF	
			Right eye		Left eye		Right eye		Left eye	
	*n*	%	*n*	%	*n*	%	*n*	%	*n*	%
Normal	46	53.5	23	31.1	24	32.4	11	23.4	13	28.9
Abnormal	40	46.5	51	68.9	50	67.6	36	76.6	32	71.1
**Total**	**86**	**100**	**74**	**100**	**74**	**100**	**47**	**100**	**45**	**100**

*Source*: Moodley A, Rae W, Bhigjee A, et al. Early clinical and subclinical visual evoked potential and Humphrey's visual field defects in cryptococcal meningitis. PloS One. 2012;7:e52895. PMID: 23285220, http://dx.doi.org/10.1371/journal.pone.0052895

VEP, visual evoked potential; HVF, Humphrey's visual fieldz

## Mechanisms implicated

Rex's classification of visual loss was time based.^6^ He suggested that early visual loss was a result of optic nerve infiltration/inflammation and occurred within 6 days of the onset of meningitic symptoms, whereas late visual loss occurred a few weeks into the infection and was the result of optic disc oedema from raised intracranial pressure (papilloedema). Remarkably, the rapid visual loss group in Rex's series had elevated CSF pressure (90%), thickened optic nerves on computed tomography (CT) scan (22%) and symmetrical visual loss (93%). The visual loss occurred before or soon after initiation of antifungal therapy and was severe and permanent. The slow visual loss group did not differ much, having elevated CSF pressure (83%), thickened optic nerves (22%) and symmetrical visual loss (93%). As to whether there was dilatation of the peri-optic CSF space or thickening of the optic nerve itself was not defined on CT scan for both groups in Rex's series. So, apart from the tempo of presentation, a clear distinction between these groups does seem artificial. We too have previously shown that raised intracranial pressure is common in CM-induced visual loss (69%), and that papilloedema was present in only 25%; but, in addition, we have shown that on MRI there is no difference between the optic nerve sheath diameter in patients with CM and that of a normal control group, regardless of CSF pressure.^7^ None of the optic nerves demonstrated post-contrast enhancement either, reflecting a poor inflammatory response. Evidence for a third mechanism of optic nerve dysfunction was compelling.

Subsequent reports, as discussed below, have supported or refuted the findings of Rex with evidence for and against the optic neuritis and papilloedema models. However, his work certainly laid down the foundation for investigation into Cryptococcus-induced visual loss; and in fact much of our current understanding has resulted from his original observations.

Recovery of vision has always been documented as poor. Drug treatment alone is insufficient as demonstrated by Graybill et al. where steroids alone were ineffective but serial lumbar punctures and reduction of CSF pressure were more successful.^8^ In Torres's meta-analysis of rapid and slow visual loss cases, the outcome was generally poor when only the underlying CM was treated and not the raised intracranial pressure.^9^

### The papilloedema mechanism

**FIGURE 2:**
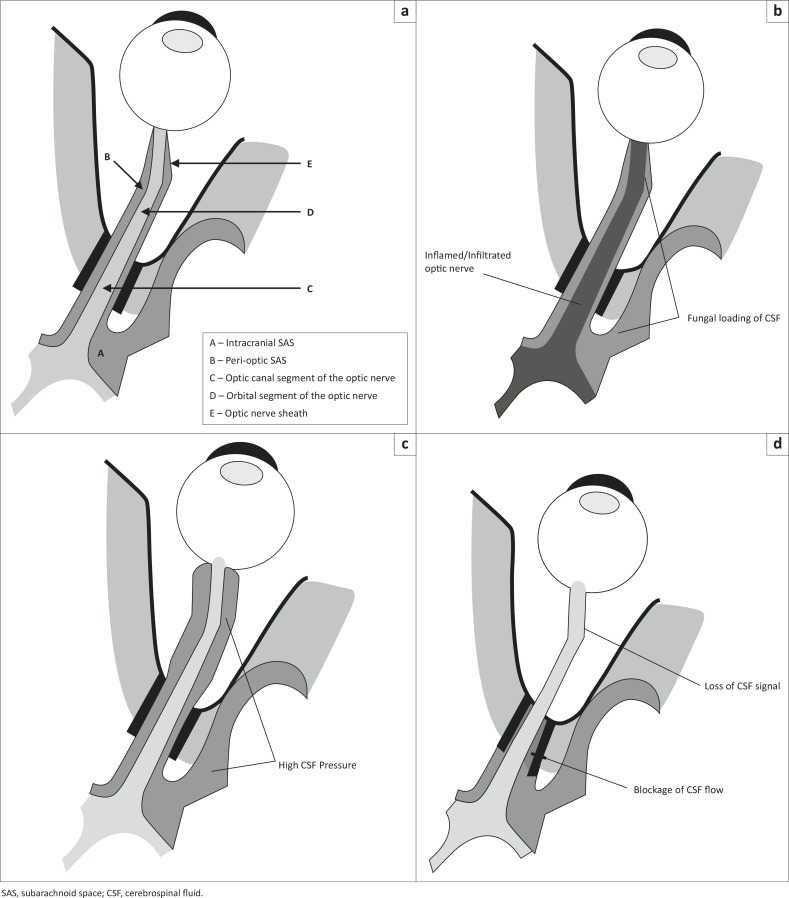
Proposed mechanisms involved in Cryptococcus-induced visual loss. (a) Normal, (b) inflammation/infiltration, (c) papilloedema and (d) Compartment syndrome.

Raised intracranial pressure in CM is well documented.^10,11^ CSF outflow obstruction caused by plugging of the arachnoid granulations by the organism and/or polysaccharide capsule is postulated to result in the elevated intracranial pressure (Figure 2c).^10,12^ Good support for obstruction at the arachnoid villi has come from Loyse et al. who demonstrated histopathologically that fungal loading (high fungal burden) occurs within the arachnoid villi and is positively correlated with elevated intracranial pressure.^12^ Bicanic et al. have shown that higher fungal burden and higher cryptococcal antigen titres are associated with higher intracranial pressure and have therefore recommended early and aggressive fungicidal treatment with lowering of intracranial pressure by either serial lumbar punctures or lumbar drainage to lower morbidity and mortality in patients with CM.^13^ In 1993, Garrity et al. performed optic nerve sheath fenestrations in two patients with visual loss and papilloedema.^14^ Following the procedure, both patients had improved vision from lowering of intracranial pressure. Cryptococcal organisms were present in the dural sheaths of both patients. At autopsy of one of the patients, patency of the sheath fenestration was still present.

The evidence for visual loss resulting from raised intracranial pressure and papilloedema, and the benefit from CSF pressure lowering either by serial lumbar punctures,^8,10,15,16,17^ acetazolamide,^17,18^ lumbo-peritoneal (LP) shunt, lumbar drain,^18,19,20^ ventriculo-peritoneal (VP) shunt^10,21^ and optic nerve sheath fenestration^14,22^ are well documented, but unfortunately mostly anecdotal. Comparative studies between surgical lowering of intracranial pressure and drug-only therapy to prevent or reverse visual loss in CM have not been done. Lowering of the raised intracranial pressure is shown to improve the overall prognosis of CM and therefore cannot be ethically withheld in a randomised controlled trial. Pharmaceutical approaches alone to control raised intracranial pressure in CM have not been shown to be effective. Surgically invasive methods to decrease intracranial pressure in CM also carry their own risks such as over drainage, shunt infection, distal catheter migration and need for shunt revision.^23^ VP shunts are associated with lower risk of shunt obstruction and revision than LP shunts and are therefore recommended when serial lumbar punctures are ineffective or not an option.

CT and MRI scans show normal ventricular size in most cases of CM despite profoundly elevated CSF pressure. Presumably the equivalent pressures between the intraventricular fluid and the CSF surrounding the brain and the paucity of intraventricular fungal elements prevent ventricular dilatation, unlike tuberculous meningitis where hydrocephalus is often encountered from blockage at the Sylvian aqueduct or foramina of Lushka and Magendie.^10^ Raised intracranial pressure and fungal loading are common and well described in CM patients, but inflammation is minimal if at all, regardless of HIV coinfection. The frequent finding of an acellular CSF in CM despite markedly elevated CSF pressure is a case in point. The significance of raised intracranial pressure cannot be underestimated in visual loss, and perhaps optic disc swelling and optic nerve infiltration/inflammation are secondary or co-occurrences. Reports of raised intracranial pressure-related visual loss are many in the literature, and the benefit of early lowering of intracranial pressure in reversing blindness in Cryptococcus-induced visual loss is well documented.^15,19,21,24^

### The optic nerve infiltration/inflammation mechanism

Evidence for optic nerve infiltration by *C. neoformans* has come from case reports only. Lipson et al*.* first described two cases of AIDS-associated cryptococcal arachnoiditis resulting in bilateral visual loss secondary to an optic neuropathy (Figure 2b).^25^ Ofner's claim of optic nerve infiltration in a patient with visual loss and elevated intracranial pressure was not robust.^26^ Histology obtained from the optic nerve sheath showed fungal infiltration with inflammation, but optic nerve infiltration was only presumed. Histological evidence of cryptococcal infiltration of the intracanalicular segment of the optic nerve with associated necrosis was provided by Cohen et al. in 1993^27^ and further supported by a histopathological case reported by Corti et al. in 2010.^28^ Corti's case also showed a perineuritis, but in addition showed optic nerve infiltration by the fungus. By inference, Hoepelman^29^ and Seaton^30^ suggested that corticosteroids could only play a beneficial role in Cryptococcus-induced visual loss by reducing the optic nerve inflammation so induced by the organism. Further support for an optic neuritis model has come from De Schacht's report of a 26-year-old CM patient who developed an immune reconstitution illness with bilateral blindness after starting antiretroviral therapy.^31^ Supposedly, the exaggerated optic nerve inflammation secondary to fungal infiltration caused the bilateral blindness. Unfortunately, such case reports in the literature are scanty and the evidence for optic nerve infiltration is mostly speculative.^32^ In our cohort of patients, optic nerve infiltration was uncommon, as evidenced by the lack of nerve signal changes and enhancement on MRI, and the dissimilar magnetic resonance diffusion parameters to that of optic neuritis.^7^ Optic nerve infiltration possibly results from direct cryptococcal invasion from the peri-optic CSF, or perhaps develops from retrograde extension of the meningo-encephalitis from the thalamus and other diencephalic structures that seem particularly susceptible to cryptococcal infiltration. The common finding of pseudocysts and dilated Virchow-Robin spaces in these regions supports this assertion.

### The optic nerve sheath compartment mechanism

We have reported a case that demonstrates the strong likelihood of ONSCS which we propose as a probable third mechanism of optic nerve dysfunction (Figure 2d). During elevated CSF pressure, there was loss of the peri-optic CSF signal on T2 MRI and return of the CSF signal following lowering of CSF pressure.^33^ The stasis of contrast-filled CSF at the mid-orbital segment of the optic nerve sheath suggests complete plugging of the peri-optic space by cryptococcal fungal elements. We further postulate that a large pressure gradient resulted from blockage between the significantly elevated intracranial pressure within the intracranial subarachnoid space (SAS) and the pressure of the proximal peri-optic CSF space. An ONSCS thus followed, causing optic nerve compression, axoplasmic stasis and ischaemia. Optic nerve dysfunction ensued with visual blurring and visual loss.

A subsequent case of a 33-year-old HIV-infected patient with bilateral blindness from CM and elevated CSF pressure (> 50 cm CSF) also demonstrates this phenomenon. In addition to the blockage within the optic canals bilaterally, dilatation of the peri-optic CSF space ahead of the obstruction is visible on the left side (Figure 3). Notably, this case showed obstruction within the optic canal, unlike the previous case that showed mid-orbital obstruction. After lowering of the CSF pressure, there is return of the CSF to the orbital peri-optic space. We postulate that, following the blockage at the optic canal level, CSF from the orbital peri-optic space is drained by the peri-optic lymphatics and hence there is loss of the CSF signal on the T2 high-resolution scan. This is more plausible than loss of the CSF signal prior to CSF pressure lowering being the result of fungal loading alone, as the interval between the two scans was only 11 days and much too soon for all the fungal elements to clear from that space.

**FIGURE 3:**
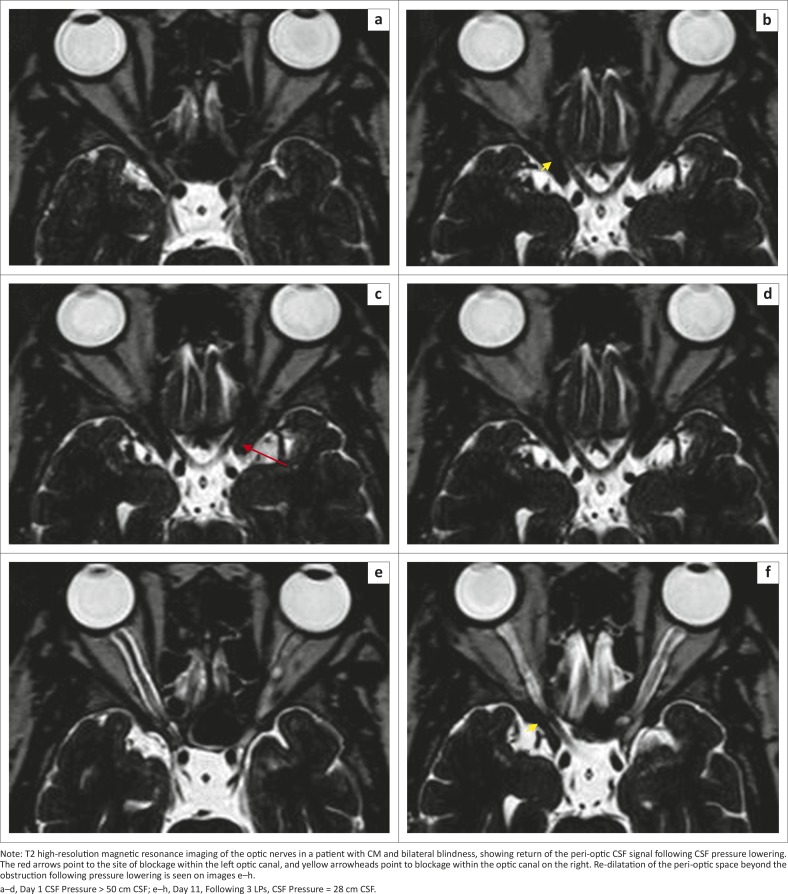
(a–h), Illustrative case of optic nerve sheath compartment syndrome.

**FIGURE 3 (Continues...):**
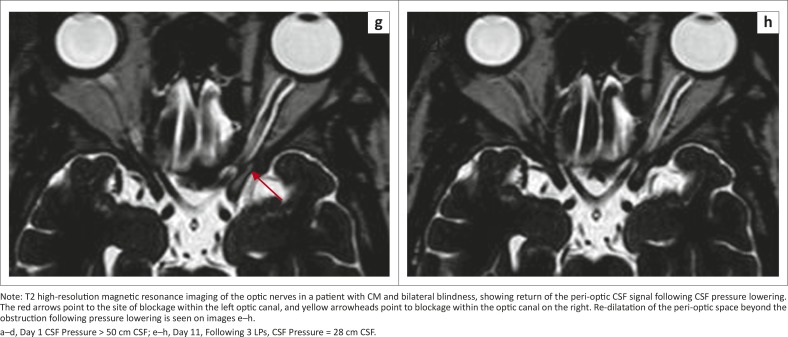
(a–h), Illustrative case of optic nerve sheath compartment syndrome.

Cohen's histological description of intracanalicular necrosis of the optic nerve and obliteration of the intracanalicular peri-optic space by fungal loading provides the only credible histopathological evidence of a clear compartment syndrome in CM-induced visual loss.^27^

Killer et al. have shown by electron microscopy that the peri-optic SAS is not occupied by CSF alone but also by a network of trabeculae, septae and pillars comprising fibroblasts and blood vessels.^34^ They provide histological evidence that the peri-optic SAS narrows in the mid-orbital segment where the delicate trabeculae change into broader septae and stout pillars that subdivide the SAS into compartments. The SAS within the intracanalicular segment is extremely narrow and consists of pillars and trabeculae only. Hence the potential sites of blockage to CSF flow are the mid-orbital and the intracanalicular segments of the peri-optic SAS.

Killer et al. also suggest that the varying anatomy of these subarachnoid trabeculae, septae and pillars between the two optic nerves account for the asymmetrical papilloedema in idiopathic intracranial hypertension (IIH).^35^ Asymmetrical pressure is transmitted to the laminar cribrosa of the two optic nerves. On the side with more trabeculae, septae and pillars, a lower pressure is transmitted to the optic nerve head and hence little or no papilloedema ensues. We have expanded on this theory in CM, where, in addition to raised intracranial pressure, there is loading of the peri-optic SAS with fungal elements (the organism and fragments of the polysaccharide capsule). CM is a pauci-inflammatory disorder, and hence fungal loading rather than inflammatory cell accumulation occurs. Fungal loading in the peri-optic CSF space has been shown by histology of the optic nerve sheath during optic nerve sheath fenestration.^22^ Sequestration of CSF in the immediate retrobulbar space is not evident, and the contribution made by the fungal clumping needs exploration. We have not been able to demonstrate optic nerve sheath dilatation nor optic nerve thickening in the setting of CM with or without papilloedema and regardless of CSF pressure measured at the lumbar level.^7^ It is conceivable that the retrobulbar segment of the nerve is subjected to toxic byproducts from the fungi, venous stasis, ischaemia from vascular compromise and axoplasmic stasis from mechanical compression. We propose therefore that these findings suggest axoplasmic stasis, mitochondrial dysfunction and ischaemia of the axons which develop from compression at the site of blockage. The end result is optic disc swelling that occurs in only 25% of CM patients, despite raised intracranial pressure occurring in 69% – 90% of CM patients. The blockage at the mid-orbital or intracanalicular segments from raised pressure and fungal elements creates compartmentalisation between the peri-optic SAS and the intracranial SAS. We propose that the raised pressure and fungal loading cause apposition of the trabeculae, septae and pillars against each other, creating a block by a valve-like mechanism. When intracranial pressure is then lowered, reopening of the channels between the trabeculae and septae occurs, and re-establishment of CSF flow to the peri-optic space.

Furthermore, the lack of optic nerve sheath dilatation and optic nerve signal changes on MRI make papilloedema and optic nerve infiltration less likely to be the only pathogenic mechanisms in CM-induced visual loss. Magnetic resonance diffusion studies do not support optic neuritis as an early cause of visual loss in CM.^7^ The co-occurrence of elevated CSF pressure, swollen optic disc and visual loss was in 15.4% of our cohort, visual loss and swollen discs in 17.3%, and visual loss and elevated pressure in 26.9%. Whilst visual loss was documented in 34.6% and elevated pressure was recorded in 69%, clearly disc swelling alone either from optic nerve infiltration or papilloedema was insufficient to account for all cases of visual loss. Elevated CSF pressure with an additional compromise of optic nerve function seems likely – not from axoplasma stasis at the lamina cribrosa but compression upstream. We postulate that this compression results from ONSCS. The steep pressure gradients at the optic canal or mid-orbital level and fungal elements trapped by subarachnoid trabeculae cause a functional block that reverses with pressure lowering. We prefer the term ONSCS to explain the above pathogenesis, which is in line with Killer's explanation of asymmetrical papilloedema in IIH,^35^ but different to the optic nerve compartmentation he described in optic neuritis where CSF was trapped in the bulbar peri-optic space, causing disc swelling.^36^ Our use of the term is also different from Orgul's description of optic nerve compartment syndrome.^37^ Orgul uses the term to describe compartmentalisation in glaucoma where the slit-like pores in the lamina cribrosa cause venous congestion and constriction of the nerve fibre bundles.

## Management of visual loss

Early screening of vision in patients with CM is imperative. Screening should involve proper Snellen chart assessments with pinhole correction if required. Baseline documentation of visual acuity with weekly documentation during the first 4 weeks and bimonthly thereafter until the maintenance phase is complete is essential and should become standard practice. When there is doubt, VEP can be done to detect subtle and even preclinical optic nerve disease. HVF is useful and should be done at initiation of treatment and repeated 4 weeks later when cognition improves with treatment. Field defects are possible, even with intact visual acuity.

With raised intracranial pressure being the predominant mechanism by which visual loss occurs, it is prudent to address this complication in CM. Antifungal therapy alone is insufficient. Early and aggressive lowering of intracranial pressure not only improves the overall prognosis of CM but also definitely prevents, alleviates and reverses visual loss.^13^ The benefit of intracranial pressure lowering is well documented by using serial lumbar punctures, lumbar drains, VP shunts and optic nerve sheath fenestration.^8,10,11,15,16,20,21,22^ Reports of reversal of visual loss from CSF pressure lowering are encouraging. Medical management alone of raised intracranial pressure in lowering CSF pressure and thereby improving vision has been less satisfactory.^38^ The latest recommendations by the Southern African HIV Clinicians Society (2013) are to remove 10 mL – 30 mL of CSF if opening pressure is > 25 cm CSF and daily LP's until symptoms of raised intracranial pressure settle.^39^ Evidence for the benefit of corticosteroids and nonsteroidal anti-inflammatory drugs (NSAIDs) to decrease optic nerve inflammation and thus improve vision has been anecdotal at best and is counter-intuitive, considering the pauci-inflammatory state of CM.^30^

## Conclusion

**FIGURE 4:**
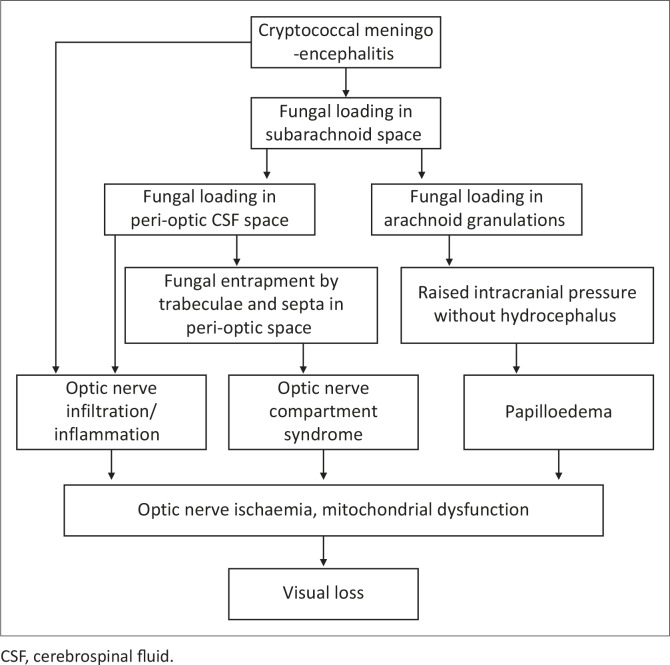
The pathogenesis of Cryptococcus-induced visual loss.

The major limitations of our case series have been the lack of long-term follow-up and the exclusion of patients with depressed levels of consciousness. However, we feel that the data from this cohort is compelling and certainly contributes to the improved understanding of Cryptococcus-induced visual loss. Visual loss in CM is common and varies from mild loss to no light perception. Bilateral involvement is usual and occurs at any time during the illness, regardless of drug therapy. Electrophysiology shows early and subclinical optic nerve dysfunction in CM. Three mechanisms seem to operate in the pathogenesis of CM-induced visual loss: (1) papilloedema, (2) optic nerve infiltration/inflammation and (3) ONSCS (Figures 2 and 4). Optic nerve infiltration/inflammation does occur but infrequently and is either a manifestation of the meningo-encephalitis that extends to the optic nerve by continuous spread from the diencephalon or a result of direct infiltration of fungi from the peri-optic CSF space. Raised intracranial pressure plays an important role in visual loss with or without papilloedema. When papilloedema and optic nerve infiltration are not demonstrable, raised intracranial pressure causes optic nerve dysfunction and visual loss, presumably by ONSCS. Fungal loading and obstruction of the peri-optic CSF space compartmentalising the intra-orbital peri-optic SAS from the intracranial SAS is probably the key mechanism but needs further investigation. Our understanding of Cryptococcus-induced visual loss has improved since Rex's initial contribution to the field. However, further studies are eagerly awaited – and preferably those that focus on CSF flow, the peri-optic and intracranial SAS compartments and the impact of pressure lowering measures on visual acuity.
